# Systemic Administration of Polyelectrolyte Microcapsules: Where Do They Accumulate and When? In Vivo and Ex Vivo Study

**DOI:** 10.3390/nano8100812

**Published:** 2018-10-10

**Authors:** Nikita A. Navolokin, Sergei V. German, Alla B. Bucharskaya, Olga S. Godage, Viktor V. Zuev, Galina N. Maslyakova, Nikolaiy A. Pyataev, Pavel S. Zamyshliaev, Mikhail N. Zharkov, Georgy S. Terentyuk, Dmitry A. Gorin, Gleb B. Sukhorukov

**Affiliations:** 1 Remote Controlled Theranostic Systems Lab, Saratov State University, Saratov 410012, Russia; nik-navolokin@yandex.ru (N.A.N.); gsv0709@mail.ru (S.V.G.); gmaslyakova@yandex.ru (G.N.M.); vetklinikanew@mail.ru (G.S.T.); gorinda@mail.ru (D.A.G.); 2 Scientific Research Institute of Fundamental and Clinical Uronephrology, Saratov Medical State University, Saratov 410000, Russia; allaalla_72@mail.ru (A.B.B.); olgabess@yandex.ru (O.S.G.); zuev.viktor.sgmu@gmail.com (V.V.Z.); 3 Biophotonics Laboratory, Skoltech Center for Photonics and Quantum Materials, Skolkovo Institute of Science and Technology, Moscow 121205, Russia; 4 Laboratory of Pharmacokinetics and Targeted Drug Delivery, Medicine Institute, National Research Ogarev Mordovia State University, Saransk 430005, Russia; pyataevna@mail.ru (N.A.P.); zamyshlyaev@gmail.com (P.S.Z.); mikhail.zharkov.92@mail.ru (M.N.Z.); 5 School of Engineering and Materials Science, Queen Mary University of London, London E1 4NS, UK

**Keywords:** polymer microcapsules, magnetite nanoparticles, biodistribution, magnetic resonance imaging, electron spin resonance spectroscopy, histological examination, atomic absorption spectroscopy, intravenous injections

## Abstract

Multilayer capsules of 4 microns in size made of biodegradable polymers and iron oxide magnetite nanoparticles have been injected intravenously into rats. The time-dependent microcapsule distribution in organs was investigated in vivo by magnetic resonance imaging (MRI) and ex vivo by histological examination (HE), atomic absorption spectroscopy (AAS) and electron spin resonance (ESR), as these methods provide information at different stages of microcapsule degradation. The following organs were collected: Kidney, liver, lung, and spleen through 15 min, 1 h, 4 h, 24 h, 14 days, and 30 days after intravenous injections (IVIs) of microcapsules in a saline buffer at a dosage of 2.5 × 10^9^ capsule per kg. The IVI of microcapsules resulted in reversible morphological changes in most of the examined inner organs (kidney, heart, liver, and spleen). The capsules lost their integrity due to degradation over 24 h, and some traces of iron oxide nanoparticles were seen at 7 days in spleen and liver structure. The morphological structure of the tissues was completely restored one month after IVI of microcapsules. Comprehensive analysis of the biodistribution and degradation of entire capsules and magnetite nanoparticles as their components gave us grounds to recommend these composite microcapsules as useful and safe tools for drug delivery applications.

## 1. Introduction

Novel drug delivery systems have been in the focus of research in bio-nanotechnology in the past decades. Substantial progress in biomedicine and applied chemistry resulted in the development of reasonably effective delivery systems aimed to bring bioactive compounds via chemical targeting to a particular site of the body, organs and tissues. The major demand in the area is how to direct the vesicles to the tumor site, which remains challenging due to side effects. Most of the elaborated delivery systems are “passive” in terms of external navigation and control over their delivery. Recent developments in nanobiotechnology have made an essential contribution, as they deal with fabrication of constructs enabling multimodal functioning, carrying bioactive molecules and being visible and addressable externally. Logically, if a delivery system represents a sort of vesicle in order to make it visible and addressable, these vesicles should also incorporate nanoparticles such as magnetite nanoparticles, which can be seen by full-body imaging techniques such as magnetic resonance imaging (MRI).

Among the technologies available so far for drug delivery, in general, there are a limited number of techniques enabling multifunctionality. Multifunctionality in this particular context is the combination of the following: Ability to carry bioactive substances, navigate to a specific site, be biodegradable after deploying the cargo, and susceptible to external activation and visualization. Obviously, the components of these multifunctional delivery systems should be responsive to local media or external stimuli. Use of externally guided nanostructured carriers for drug delivery is a promising method in bio-nanotechnology, which can be used in areas such as diagnostics of tumors [1,2] for enhanced contrast at MRI visualization [3,4], targeted delivery of drugs to specific organs and tissues [5,6,7] and for magnetic hyperthermia of tumors [8,9,10]. This research has been undertaken to illustrate the promise of addressed delivery to particular sites in the body with the help of magnetic nanoparticles externally navigated with a magnetic field [11], which might also work as an accomplishing method with biological targeting performed by conjugation of nanoparticles with tumor-specific antibodies, followed by accumulation of nanoparticles in the targets [12].

In light of the development of multifunctional delivery systems more than a decade ago, the principles of layer-by-layer (lbl) assembly were applied to construct micron and submicron sized delivery systems, where various components can be simply tailored via incorporation of responsive and charge species as shell components of the capsules [13].

These capsules, proposed as delivery systems a while ago, were intensively studied mostly for their physical and chemical properties. At present, these capsules can be made of a defined size in a range from about 100 nm to several microns, contain various bioactive molecules including proteins, nucleic acids as well as small molecules, and can be externally addressed via a magnetic field, light or ultrasound [14,15]. These capsules can be taken up by various cells types, including endothelial cells, mesenchymal stem cells, microphages, neuroblastoma and others [16,17,18]. The mechanism of cell uptake is relevant to endocytosis and, as most reports have demonstrated, there is a minimal or absent effect on cell viability. Various cell types showed high percentages of survival at an excess of capsules per cell ratio. Capsule degradation inside the cells varied from a few to up to 24 h, depending on the cell type and capsule composition. The release of encapsulated materials inside the cells can be gradual or triggered externally if light is used to open the capsules while inside the cells and releases the cargo to cytoplasm [19,20,21,22,23]. 

Despite the intense study on capsule properties and their interaction with cells, there is a lack of reported data on how the capsules would behave if administered systemically in vivo. So far, there have been few reports on lbl capsules administered in vivo either via subcutaneous injection or nasal gavage [24,25,26,27]. There are attempts for MRI imaging of iron oxide modified capsules. However, there are no systematic studies of lbl capsule distribution in organs or at what time point they are accumulated in a particular part of the body once they are introduced systemically. 

The aim of this study was to examine capsule distribution in vivo upon systemic delivery via the tail and to explore major organs, such as the liver, lung, heart, spleen, and kidney for capsule presence at different time points in rats. Particular attention was given to evaluating for how long the capsules and debris of capsule degradation were present in these organs and what their degradation times were. A detailed analysis of capsule fate in vivo is very complex and requires various methods for the unambiguous identification of capsules and their components. In order to facilitate capsule identification, capsules were made of biodegradable polymers to ensure their degradation was modified with magnetite nanoparticles sandwiched between the layers. Complex analysis was conducted in vivo using MRI visualization and on ex vivo samples using atomic absorption spectroscopy (AAS), electron spin resonance (ESR) for detection of iron as an element and as superparamagnetic nanoparticles, respectively, and direct histology visualization of selected organs.

## 2. Materials and Methods

### 2.1. Magnetic Microcapsule Preparation

Magnetic microcapsules were prepared using the layer-by-layer technique [28]. Poly-l-arginine hydrochloride (Parg, MW ~70 kDa), dextran sulfate sodium salt (Dex, MW ~70 kDa), and sodium chloride (anhydrous) were used without further purification and were purchased from Sigma-Aldrich GmbH, Germany. The water used in all experiments was prepared in a UVOI-1M purification system (Mediana-filter, Moscow, Russia) and had a resistivity higher than 14 MΩ•cm.

The following materials were used for the microcapsule preparation: CaCO_3_ microparticles (diameter, 4 ± 0.7 μm), poly-L-arginine and dextran sulfate sodium salt diluted in 0.15 M NaCl water solution; magnetite hydrosol (diameter, 13 ± 5 nm and zeta potential, −31 ± 9 mV measured by the DLS method) (Figure S1). In this work, the method described previously by Massart was used for iron oxide nanoparticle synthesis [29]. Synthesis was carried out using the setup described in reference [30].

**Figure 1:**
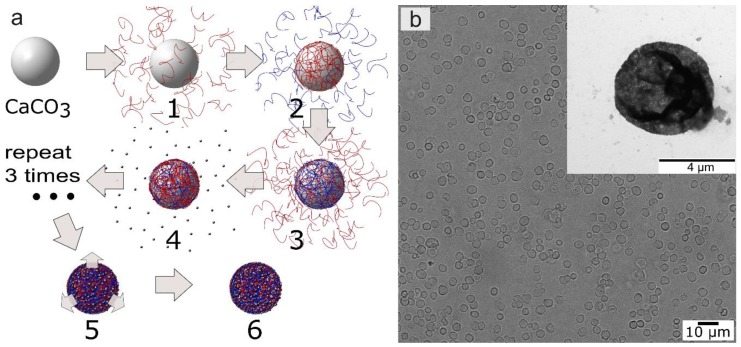
(**a**) Scheme of microcapsule preparation: 1, 3–adsorption of Parg, 2–adsorption of Dex, 4–adsorption of magnetite nanoparticles (MNP)s, 5–dissolution of core, 6–magnetic polyelectrolite microcapsule. (**b**) Optical and transmission electron microscopy (TEM) (inset figure) images of biodegradable microcapsules containing magnetite MNPs.

The nanocomposite polyelectrolyte shells were formed on the surface of calcium carbonate microparticles. Polyelectrolyte shells were prepared by lbl assembly technique via alternate treating microparticles in solutions of oppositely charged polyelectrolytes and nanoparticles. These were poly-l-arginine (Parg), dextran sulfate sodium salt (Dex) and magnetite nanoparticles (MNPs). The consecutive adsorption of Parg and MNPs was repeated three times and finally, the capsules had the following composition: Parg/Dex/(Parg/MNPs)_3_/Parg/Dex (Figure 1a). The microcapsule with each freshly deposited layer was washed two times with deionized water before starting the next deposition step. Optical and transmission electron microscopy (TEM) images of magnetic microcapsules are presented in Figure 1b. The concentration of the microcapsules was determined using a hemocytometer and it was of the order of 5 × 10^8^ mL^−1^.

The polyelectrolyte composites containing this type of magnetite nanoparticles exhibit the superparamagnetic behavior which was shown on the planar polyelectrolyte composite coatings. [31]. Variation of the volume fraction of inorganic nanoparticles led us to control the physical properties of microcapsule shells as well as MRI contrast. Contrast enhancement of magnetic microcapsules increases with increasing average distance between magnetite nanoparticles in the shell [32]. In agreement with the analyses of some already published articles, we can conclude that size stability of such type of microcapsule in vitro is very high [33].

### 2.2. Dynamic Light Scattering, Atomic Force Microscopy, and Transmission Electron Microscopy

The measurements of the zeta-potential and size distribution of nanoparticles were performed using a Zetasizer Nano-ZS instrument (Malvern Instruments Ltd., Malvern, UK). 

Atomic force microscopy (AFM) images of microcapsules were obtained with a Ntegra Spectra microscope (NT-MDT, Zelenograd, Moscow, Russia) in tapping mode. For image acquisition, NSG10 probes from NT-MDT with a resonant frequency of about 220 kHz, a force constant of 3.1–37.6 N/m and a tip curvature below 10 nm were used. Samples were prepared by drying a drop of the microcapsule suspension on the cover glass slide surface. All image processing was done with Gwyddion software [34].

Transmission electron microscopy (TEM) images were obtained using a Libra-120 transmission electron microscope (Carl Zeiss, Jena, Germany) operating at 120 kV. The samples were prepared by deposition of a capsule suspension onto a formvar film supported by the copper grid. 

### 2.3. Animal Study. Ex Vivo Organ Preparation

Animal experiments were performed in accordance with the University’s Animal Ethics Committee and the relevant international agency [35] in Core Facilities of Saratov State Medical University. In experiments, 42 white outbred male rats weighing 200 ± 20 g were used. Thirty-six rats were intravenously injected with a single dose of a microcapsule suspension dispersed in physiological saline at 2.5 × 10^9^ capsules per kg. Then, the animals were randomly divided into 6 groups of 6 rats in each group; the control group consisted of 6 rats. The duration of the experiment was different in different groups; i.e., the lifetime of the animals after administration of the microcapsules was 15 min, 1 h, 4 h, 24 h, 7 days and 30 days. These time intervals were chosen as they are most frequently used for the biodistribution study [36]. 

An MRI study was performed and after that, the animals were decapitated. The following organs were collected: Kidney, liver, lung, and spleen at the indicated time points after microcapsule administration. The time dependence of microcapsule and iron distribution inside the rats was investigated by histological examination, AAS and ESR.

### 2.4. Magnetic Resonance Imaging

Magnetic resonance imaging in vivo was performed using a Philips Achieva 1.5T high field MRI scanner with a phased array coil. Immobilization of animals was carried out for 60 min in the supine position with the fixation of the limbs. Zoletil 50 (Virbac, France) was administered intramuscularly at a dose of 40 µg/kg for anesthesia. T1 and T2-weighted quick “Spin Echo” protocols (Turbo Spin Echo–TSE), and T1-weighted “Fast Field Echo” (is equal to the “Gradient Echo”) were used. The presence of contrast agents in the test object which mainly reduces the longitudinal relaxation time T1 (substances containing gadolinium, for example, gadobutrol [37]), in the tissue causes a hyperintense signal on T1-weighted images (lighter staining). Contrast agents which mainly reduce the transverse relaxation time T2 (iron oxides) cause a hypointense signal on T2-weighted images. After in vivo MRI study, the animals were decapitated.

### 2.5. Histological Examination

The sampling of internal organs (spleen, liver, kidneys, lungs, and heart) for morphological studies and determination of microcapsule accumulation were conducted after removing the animals from the experiment. Samples of internal organs were fixed in a 10% solution of buffered neutral formalin for morphological examination and subjected to standard wiring alcohol. The standard histological techniques with hematoxylin and eosin staining were used.

The capsules were counted in 10 fields of vision in each section of organ, but not less than in 3 sections, with an increase of 774 on Microvizor medical of transmitted light mVizo-103 (LOMO, St. Petersburg, Russia). The standard magnification allows one to obtain more objective data and to compare them, so it will be possible to study the dynamics of microcapsule movement and determine the time points for the accumulation of microcapsules in the organs.

### 2.6. Atomic Absorption Spectrometry

A Thermo Scientific iCE 3500 instrument (Thermo Scientific, Bartlesville, OK, USA) was used for the quantitative determination of iron in the tumor. The operating principle of the method is based on the transfer of elements defined in the atomic state. Fiery atomizer was used in the work. The element concentration was determined by the intensity of the light absorption with the characteristic wavelength of atomic vapor of the element. The wavelength was 248.3 nm for Fe, the slit width was 0.2 nm, and the lamp current was 75%. A hollow cathode lamp was used as the light source. A standard sample of metal ions (GSO 7330-96, Saint Petersburg, Russia) was used for the calibration of the spectrometer.

### 2.7. Quantitative Magnetite Content Analysis via Electron Spin Resonance (ESR) Spectroscopy

In order to evaluate microcapsules presence in organs, a quantitative magnetite content analysis of ex vivo samples was performed using the ESR method according to the procedure described by Chertok et al. [38] with modification given below in this section. 

The modified procedure of ESR spectroscopy in this study is based on the recording of ferromagnetic resonance spectra reflecting the magnetite content in a specimen. The paramagnetic peaks of the ionized iron become totally smooth and really cannot influence the intensity and other characteristics of the signal. Thus, the endogenous iron does not interfere with determination of ferromagnetic capsules. Furthermore, the constant distribution of magnetite over the capsules and the uniform environment for the incorporated magnetite (the polyelectrolytes surrounding the magnetite) guarantee that the resonance field and the spectrum forms are not different for any specimens containing microcapsules. This makes it possible to calibrate the spectrometer with in vitro specimens of a microcapsules suspension and to measure the microcapsule and magnetite content in ex vivo specimens without further corrections, including the control correction.

Before ESR analysis, pieces of extracted organs weighing 150–200 mg were dried in a vacuum oven and fixed to the quartz rod. After that, they were inserted into a recording unit (block) of the ESR spectrometer (The scheme is shown in Supporting Information (Figure S3)).

ESR spectra were obtained using a CMS 8400 X-band ESR spectrometer (Adani, Belarus) with the following parameters: Resonant frequency, ~9.2 GHz; center field, 3100 G; sweep width, 3400 G; modulation amplitude, 1 G. 

In our studies, the ESR method originally proposed in the paper [38] has been modified: ESR spectra were recorded in dry samples at room temperature (21 °C) in contrast to low-temperature (−128 °C) measurements in frozen samples used by Chertok et al. This modification makes the recording easier to use without significant loss of accuracy. The obtained calibration curve for the contain/signal relationship have proved the relation to be linear in the investigated concentration range (from 0.5 to 20 µg of magnetite per sample) for dry capsules. The calibration curve is given in SI (Figure S4).

Spectra were recorded as the first derivative of absorbed microwave power (P) versus the applied magnetic field (B) and are given as (dP/dB). The double integral of the collected spectra (∫∫(dP/dB)dBdB) is known to be proportional to the number of resonating electronic spins in a measured sample. Double integral values were obtained from spectra using the EPRCMD 4.0 program (Adani, Minsk, Belarus). 

The concentration of magnetite in the samples was obtained with the help of a calibration curve, which was made using samples with the known content of magnetite in form of a dried suspension of magnetite microcapsules of known concentration. The samples were dried in vacuum at ESR-neutral substrate and then investigated by the procedure mentioned above.

Control experiments, conducted with blood and tissue samples without any magnetite in the system, showed no ferromagnetic signal; therefore, the background correction was negligible and was not taken into account (Figure S5). It should be noted that the addition of blood and organ homogenates to the calibration samples does not alter the shape of the spectra (Figure S6) and the resonance field. This fact underpins the possibility of application of the ESR method to the quantitative analysis of magnetite microcapsules in ex vivo samples. 

## 3. Results

### 3.1. In Vivo MRI Study of Intravenously Injected Capsules

**Figure 2:**
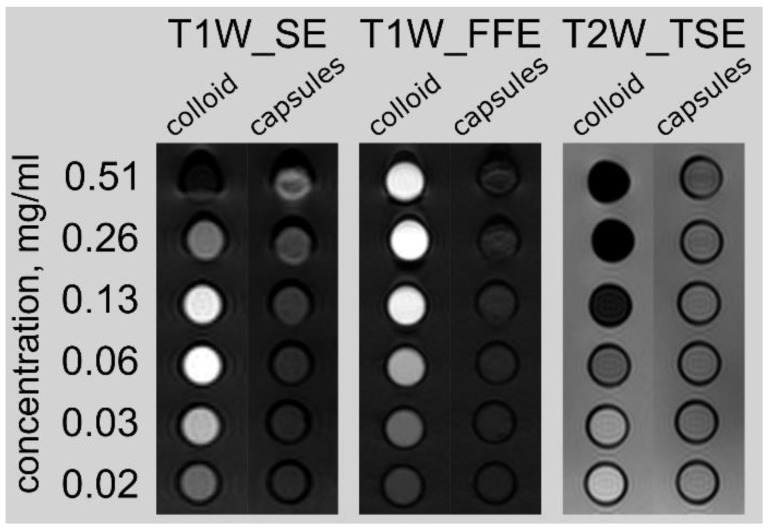
The MRI contrast of the magnetite colloid and magnetic microcapsules at a different concentrations of magnetite in the probe tubes. Different pulse sequences are presented from left to right: T1 weighted “Spin-echo” (T1W_SE), T1 weighted “Fast Field Echo” (T1W_FFE), and T2 weighted “Turbo Spin Echo” (T2W_TSE).

Initial assessment of capsule distribution was done by MRI. Magnetite nanoparticles mainly reduce the transverse relaxation time T2 and cause the darker staining of corresponding marked tissue areas on T2-weighted images (Figure 2). The microcapsules with magnetite NPs in the shell do not enhance contrast in the region of interest ROI compared to magnetite hydrosol in the same concentration, but upon enzyme degradation liberated nanoparticles enhance contrast in magnetic resonance (MR) images (Figure 2, Figure S2).

**Figure 3:**
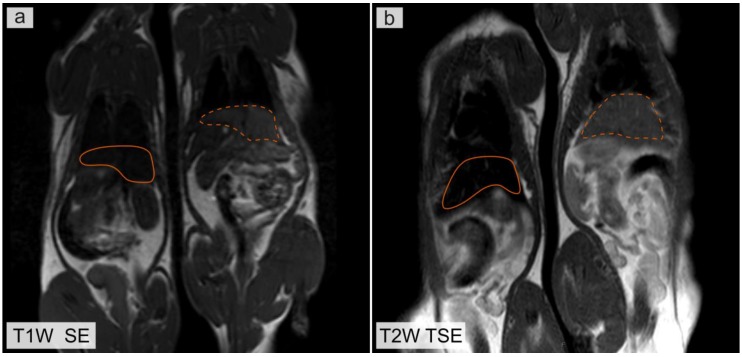
Magnetic resonance (MR) images of rats obtained 24 h after injection of a microcapsule suspension. (**a**) T1 weighted MR image. (**b**) T2 weighted image. The rat on the right is a control rat, without injection of microcapsules.

Figure 3 shows the T1 and T2 weighted MR images of rats obtained 24 h after injection of microcapsules containing magnetite nanoparticles. Solid orange lines indicate liver in a rat after microcapsule injection. Dotted orange lines indicate liver in a control rat. Magnetite distributed in liver leads to a decrease in the MR signal intensity in the region of interest (ROI) in the T1 weighted MR images (Figure 3a). This can be explained by the fact that the concentration of magnetite in the ROI was higher than 0.4 mg/mL. At high concentrations of magnetite nanoparticles, the effect of the T2 relaxation process on the measured MR signal intensity was higher than the effect of the T1 relaxation process. This leads to a decrease in the MR signal intensity in the T1 weighted images. The T2 relaxation time affects the MR signal intensity in the T1 weighted images because in clinical MRI the images are weighted by T1 and T2 but not calculated from only T1 or T2 relaxation times. Pure T1 and T2 images are not useful in clinical MRI, because the T1 and T2 values could not be applied for differential diagnosis or characterization of pathology [39]. 

The average magnetite nanoparticles concentration in the liver is less than 1 mg/mL, taking into account volume of the rat’s liver so artifacts in MR images are not observed.

According to MRI investigation, immediately after intravenous administration of microcapsules, the contrast of the region of interest is not observed. This fact is related to the high volume fraction of magnetite NPs in the microcapsules used. Microcapsules with a high volume fraction of magnetite NPs have no effect on the MR signal intensity, but upon enzyme degradation the liberated nanoparticles enhance contrast (Figure 2, Figure S2) [32]. It was established that the dependence of the MR signal on the volume fraction of magnetite was related to the interparticle distance (d) in the microcapsule shell (Figure S2). The further histological study demonstrated that the microcapsules were destroyed in the liver within 24 h and the realized magnetite nanoparticles exhibited contrast properties within 7 days (Figure 3).

Then, the distribution of the microcapsules in the organs was analyzed postmortem at selected time points by the three methods mentioned above: AAS, histology, and ESR. Appropriate qualitative and quantitative assay methods need to be established and be sensitive enough to detect the presence of microcapsules in cells and tissues. 

### 3.2. Comparative Analysis of Histology Data with AAS and ESR

The time dependence of microcapsule and iron distribution inside the internal organs of rats after intravenous injection (IVI) of biodegradable microcapsules was investigated by histological examination, AAS, and ESR. 

**Figure 4:**
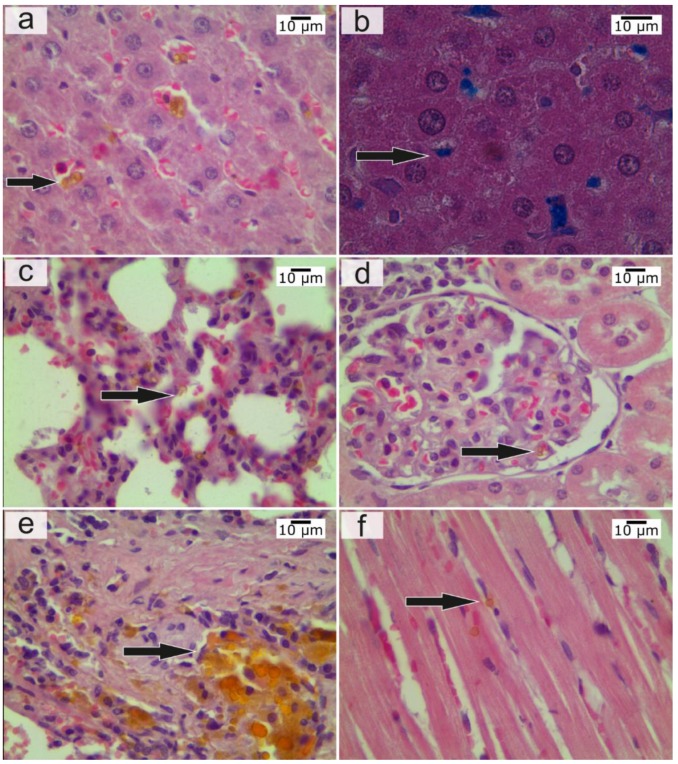
(**a**) Liver 4 h after intravenous injection (IVI) of the microcapsules—the conglomerates of the microcapsules in sinusoids. Hematoxylin and eosin (H&E), magnification 774×. (**b**) Liver 4 h after IVI of microcapsules, the conglomerates of the microcapsules were painted in blue. Prussian blue staining, magnification 1199.7×. (**c**) Lung 15 min after IVI of the microcapsules, microcapsules in capillaries of lung tissue. H&E, magnification 774×. (**d**) Kidneys 15 min after IVI of the microcapsules—the microcapsules in vascular loops of glomeruli. H&E, magnification 774×. (**e**) Spleen one day after IVI of the microcapsules—the microcapsules and magnetite were observed in spleen tissue. H&E, magnification 774×. (**f**) Heart 1 h after IVI of microcapsules—the individual microcapsules in myocardium. H&E, magnification 774×. The arrow indicates microcapsules or their clusters in the organs.

**Figure 5:**
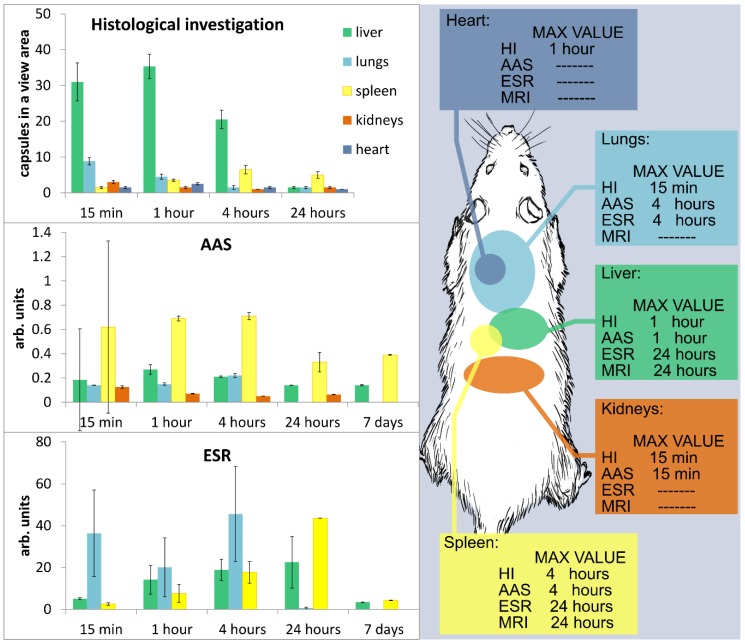
Biodistribution of magnetic microcapsules at intravenous injections. Left side: Microcapsule biodistribution data obtained by histological investigation, atomic absorption spectrometry (AAS) and electron spin resonance (ESR). Right side: maximum accumulation of microcapsules and MR signal for each organ observed.

**Liver.** At the histologic examination the content of the microcapsules increased most 1 h after IVI, while pronounced changes were noted in the form of circulatory disorders and dystrophy of hepatocytes. Four hours after IVI, the number of whole capsules decreased, but the amount of pigment increased (Figure 4a,b). At the same time, the morphological changes in the tissue were less pronounced, although there were signs of an allergic reaction with eosinophils in the lumen of the vessels. Twenty-four hours after IVI of microcapsules, the dissolution of the capsules in the Kupffer cells was noted and the hepatocytes with the release of its contents into the cytoplasm of a cell, eosinophilia was not already marked. A week later, the content of the pigment was large, and the whole capsules were only between hepatocytes or their fragments. A month later a normalized structure of the liver was observed, and the pigment was absent. According to AAS, the maximum amount of iron in the liver was observed 1 h after IVI of microcapsules. According to ESR analysis, the dynamics of magnetite distribution had a different character. Starting at a relatively small fraction at first time point of 15 min it consistently increased over 24 h and reached a maximum at the end of the first day after administration. After a week, the concentration of magnetite in the liver dropped 7-fold lower than that at the maximum (Figure 5).

**Lung.** At the histological examination, the maximum changes appeared 4 h after IVI of microcapsules in the form of pronounced congestion of large vessels, focal hemorrhages, and peribronchial eosinophilic infiltration. The severity of these changes was reduced after 24 h. A large number of capsules were also noted 15 min after IVI of microcapsules in an average amount of 8.75 ± 1.03 units in the field of view in the lumen of medium caliber vessels, between the bronchi and in the stroma (Figure 4c). One week after IVI of microcapsules, the severity of the allergic reactions increased with the involvement of the bronchi and blood vessels. One month after IVI of the microcapsules, the appearance of a large number of lymphocytes in the lungs was noted, which were in the form of widespread infiltrates located around the main bronchus. Infiltrates occupied the area in several fields of view at the lowest magnification eye field. There was pronounced hyperplasia of the muscular layer of vessels of various sizes, with ring-shaped lymphoid infiltration around blood vessels of all calibers. The area of perivascular infiltration was significantly less than that around the bronchi. According to AAS, the maximum amount of iron in the lungs was observed 4 h after IVI of microcapsule suspension. According to ESR analysis in lungs, the concentration of magnetite was highest in the early stages of ex vivo analysis (between 15 min and 4 h after administration). It was significantly decreased at the time point of 24 h, and the magnetite was not determined in the lungs in the subsequent phases of observation for week and month time (Figure 5). Unfortunately, it was not confirmed by MRI, because normal lung tissue has low proton density; therefore, magnetite-containing microcapsules were not visualized in lungs by MRI in vivo.

It should also be noted that in the lungs, a marked allergic reaction was observed during all time intervals, which was manifested by the appearance of perivascular lymphoid infiltration, and further abrupt thickening of the vessel walls, as well as more pronounced hyperplasia of bronchial lymphoid tissue, which was observed one month after the intravenous administration of the capsules. The absence of marked toxicity in the internal organs after IVI of microcapsules are consistent with our data obtained earlier [32].

**Spleen.** At the histological examination the maximum changes occurred 4 h after IVI of microcapsules in the form of pronounced congestion, increase in the number of microcapsules (up to 9 in the field of view in the white pulp. In red pulp, up to 4). After one day, the largest accumulation of microcapsules was noted, and there were also indirect signs of their degradation (Figure 4e). An important fact is that the signs of degradation were observed at all time intervals. After one week, single capsules were observed in the white pulp, and the pigment was located diffusely. One month after, the pigment disappeared in the red pulp and it remained in the white pulp. According to AAS, the maximum amount of iron in the spleen was observed after 4 h. According to ESR, the dynamics of magnetite accumulation in the spleen was similar to that in the liver (increasing in the first day with subsequent decreasing), but after 24 h, the concentration of magnetite in the spleen was two times higher than that in the liver (Figure 5).

**Kidneys.** At the histological examination, the maximum changes were observed 24 h after injection in the form of hemorrhages in the cortex, dilatation of capillary loops of the glomeruli, and marked degradation of the capsules with accumulation of content in the epithelium of convoluted tubules. This does not allow us to make an unambiguous conclusion: The capsules pass through the urinary filter and their contents are reabsorbed from the urine back into the tubules or capsules entered into the epithelium through the capillaries which nourish epithelium. The maximum content of whole capsules was observed in the first time points after IVI—in 15 min and 1 h (Figure 4d). After one week, the whole capsules were not detected, but the appearance of magnetite in the epithelium of convoluted tubules was noted. After one month, a normal structure of the kidney and the absence of pigment were observed. According to AAS, the maximum amount of iron in the kidneys was observed after 15 min. According to ESR analysis in kidneys the absence of magnetite may be explained as follows: The concentration of magnetite in kidneys is lower than the limit of determination even at the time of first measurement (Figure 5).

**Heart.** At the histological examination the maximum changes were observed within 1 h after administration in the form of marked edema, single diapedetic hemorrhages, swelling and necrosis of cardiomyocytes. The maximum content of the capsules was up to 2–3 in the field of view (Figure 4f). Four hours after IVI of microcapsules, the severity of changes was reduced, and capsules were not detected; moderate swelling and granular degeneration were saved to the week after the introduction and after a month, the normal structure of the myocardium was seen. AAS and ERS investigations were not carried out in the heart tissue. 

## 4. Discussion

Since the nanocomposite microcapsules are multicomponent systems consisting of three components (cationic and anionic polyelectrolytes and inorganic nanoparticles), the components have different biodegradation times and as a result, the polymer shells are degraded more quickly than the magnetite nanoparticles. Therefore, as far as the degradation of the magnetic capsule is concerned, one should consider it as a multistep process. There is an initial state before biodegradation starts, then it is likely the polymer shell degradation occurs, releasing magnetite nanoparticles which later degrade, and iron ions could be free from the nanoparticles. Such a complex process of degradation requires different complementary methods for evaluating the biodistribution of magnetic microcapsules. Morphological methods, such as histology, allow comprehensive determination of the quantitative content of capsules in sections of internal organs, time points the capsules appear there, maximum accumulation and the delocalization of whole capsules between internal organs, and, at the end, to evaluate complete capsule destruction and elimination of their components at certain time points after intravenous administration. ESR allows evaluation of the biodistribution of magnetite nanoparticles once they are intact either in capsules or released from capsules but still intact and exhibit superparamagnetic properties. MRI makes possible the visualization of magnetite nanoparticles before and after polymer shell degradation, since these peculiarities of MRI capsule degradation imaging are discussed in detail [32]. After the beginning of capsule destruction, the morphological method ceases to be adequate for further quantification, since the capsules cannot be identified any longer. Since capsule integrity is lost, one can follow the fate of magnetite nanoparticles released from the destroyed capsules by using the MRI (in vivo) and ESR (ex vivo) methods. Both of these methods can monitor magnetite nanoparticles till their degradation, leaving only iron ions, which cannot be detected any longer by MRI and ESR. Along with that, AAS allows detection of iron element biodistribution at all stages of capsule accumulation and degradation, including monitoring of iron before and after microcapsule and nanoparticle degradation. In addition, the complexity of application of these methods makes it possible to identify more clearly the time periods of biodegradation of capsules with magnetite nanoparticles in certain organs.

Comparative analysis of microcapsule biodistribution showed that a significant correlation was observed between the temporal dynamics of microcapsule content in the liver, spleen, and kidneys, according to the histological and AAS data. At histological examination, the maximum amount of magnetite microcapsules was obtained in the kidneys and lungs at 15 min, in the liver and heart at 1 h, and in the spleen at 24 h after IVI of microcapsules. At AAS the maximum amount of magnetite microcapsules was observed in the kidneys at 15 min, in the liver at 1 h, and in the spleen and lungs at 4 h after IVI of microcapsules.

ESR analysis demonstrated the magnetite distribution dynamics in various tissues. Magnetite was found in the lungs, liver, and spleen and was not detected in the kidney at the selected time point. According to ESR analysis, the maximum of magnetite accumulation developed in the lungs after 4 h, and in the liver and spleen at 24 h after intravenous administration of capsules, Biodegradation of the capsules and the release of their content begin on the first day after administration of the capsules. Additionally, the examined organs showed no presence of magnetite 30 days after IVI of microcapsules. Although the discrepancy of the results obtained by ESR and MRI could be explained by the sensitivity of both methods to magnetite nanoparticle conditions and the integrity of the microcapsule shells. According to the histological investigation, the microcapsules were degraded within 24 h, which resulted in the different behavior of MRI and ESR signals. 

The differences in the tissue distributions of magnetite and microcapsules can be explained by the action of several factors. Firstly, our study has demonstrated a significant accumulation of magnetite in the tissues with a highly developed reticuloendothelial system (liver and spleen). These data are consistent with the data from other studies [40,41], in which it was shown that nanoparticles are actively phagocytosed and accumulated in the organs with a large content of tissue macrophages.

Another factor which plays an important role in biodistribution may be the specific features of particle passage through the microcirculatory system. We assume that early-stage accumulation and the subsequent rapid decrease in the particle concentration in lungs is caused by mechanical embolization of some pulmonary capillaries with microcapsules at their first passage. The embolization is possible because the diameter of the capsules is close to the size of the pulmonary capillaries. The properties of microcirculation can also contribute to the accumulation of the microcapsules and magnetite in the liver and spleen. These organs contain open sections of the circulatory system, which makes possible the transition of the microcapsules from the blood flow to the interstitium.

## 5. Conclusions

In this study, we showed what happens with polyelectrolyte capsules modified with magnetic nanoparticles when they are systemically administered. The intravenous administration of microcapsules brings about changes in tissue morphology in most organs (kidney, heart, liver, spleen), but it is reversible and after a month, the structure of the tissue is completely restored. Whole capsules were not observed demonstrating their complete degradation, and the pigment indicating iron disappeared. Although the timeline of organ localization for capsules is coherent for other delivery systems, showing at first the accumulation in liver with traces of iron oxide seen in the spleen after 7 days, the overall picture illustrates the applicability of using these capsules for systemic delivery without visible pathological observation.

The reported data gave us more understanding about the distribution of capsules in the animal body in the dynamic time frames over hours and days and provide information about what organ and when one could expect the capsules potentially bearing bioactive cargo. The polyelectrolyte microcapsules, being on the research agenda for decades, can deliver the substance of interest to organs at a certain time after injection. Thus, the long-standing potential for application of these capsules can be further explored on particular delivery to organs. The magnetic nanoparticles have not been used here as their magnetic properties, but their addressing with magnetic field and/or electromagnetic irradiation is subject for further study where the expectations of multifunctional microcapsules to be used as multimodal drug delivery systems could be fulfilled.

## Supplementary Materials

The following are available online at http://www.mdpi.com/2079-4991/8/10/812/s1, Figure S1: Magnetite nanoparticle size distribution measured by DLS; Figure S2: Dependence of MRI contrast on interparticle distance d, illustrated by TEM images of MNPs, microcapsules after enzyme degradation and initial microcapsules.; Figure S3: The sample preparation process for ESR spectroscopy; Figure S4: Chart for mass of magnetite—ESR area under curve dependence; Figure S5: Comparison of ESR spectra of liver from animals exposed and not exposed to microcapsules; Figure S6: Comparison of ESR spectra of microcapsules suspension in the water and liver homogenate.
