# Supplementary material for: Systemic Administration of Polyelectrolyte Microcapsules: Where Do They Accumulate and When? In Vivo and Ex Vivo Study
Source: Nanomaterials (Basel). 2018 Oct 10;8(10):812. doi: 10.3390/nano8100812 (PMC6215302; doi:10.3390/nano8100812)
Supplement: Supplementary file 1 [file nanomaterials-08-00812-s001.pdf]

Supporting information

Systemic Administration of Polyelectrolyte Microcapsules: Where Do They Accumulate and When? In Vivo and Ex Vivo Study

Nikita A. Navolokin [1,2], Sergei V. German [3,1], Alla B. Bucharskaya [2], Olga S. Godage [2], Viktor V. Zuev [2], Galina N. Maslyakova [1,2], Nikolay A. Pyataev [4], Pavel S. Zamyshliaev [4], Mikhail N. Zharkov [4], Georgy S. Terentyuk [1,2], Dmitry A. Gorin [3,1] and Gleb B. Sukhorukov [5,1,*]

[1] Remote Controlled Theranostic Systems Lab, Saratov State University, Saratov, Russian Federation; nik-navolokin@yandex.ru (N.A.N.); gmaslyakova@yandex.ru (G.N.M.); vetklinikanew@mail.ru (G.S.T.);

[2] Scientific Research Institute of Fundamental and Clinical Uronephrology, Saratov Medical State University, Saratov, Russian Federation; allaalla_72@mail.ru (A.B.B.); olgabess@yandex.ru (O.S.G.); zuev.viktor.sgmu@gmail.com (V.V.Z.);

[3] Biophotonics laboratory, Skoltech Center for Photonics and Quantum Materials, Skolkovo Institute of Science and Technology, Moscow, Russian Federation; d.gorin@skoltech.ru (D.A.G.); s.german@skoltech.ru (S.V.G.)

[4] Laboratory of Pharmacokinetics and Targeted Drug Delivery, Medicine Institute, National Research Ogarev Mordovia State University, Saransk, Russian Federation; pyataevna@mail.ru (N.A.P.); zamyshlyaev@gmail.com (P.S.Z.); mikhail.zharkov.92@mail.ru (M.N.Z.)

[5] School of Engineering and Materials Science, Queen Mary University of London, London, United Kingdom

* Correspondence: g.sukhorukov@qmul.ac.uk; Tel.: +44 20 78825508

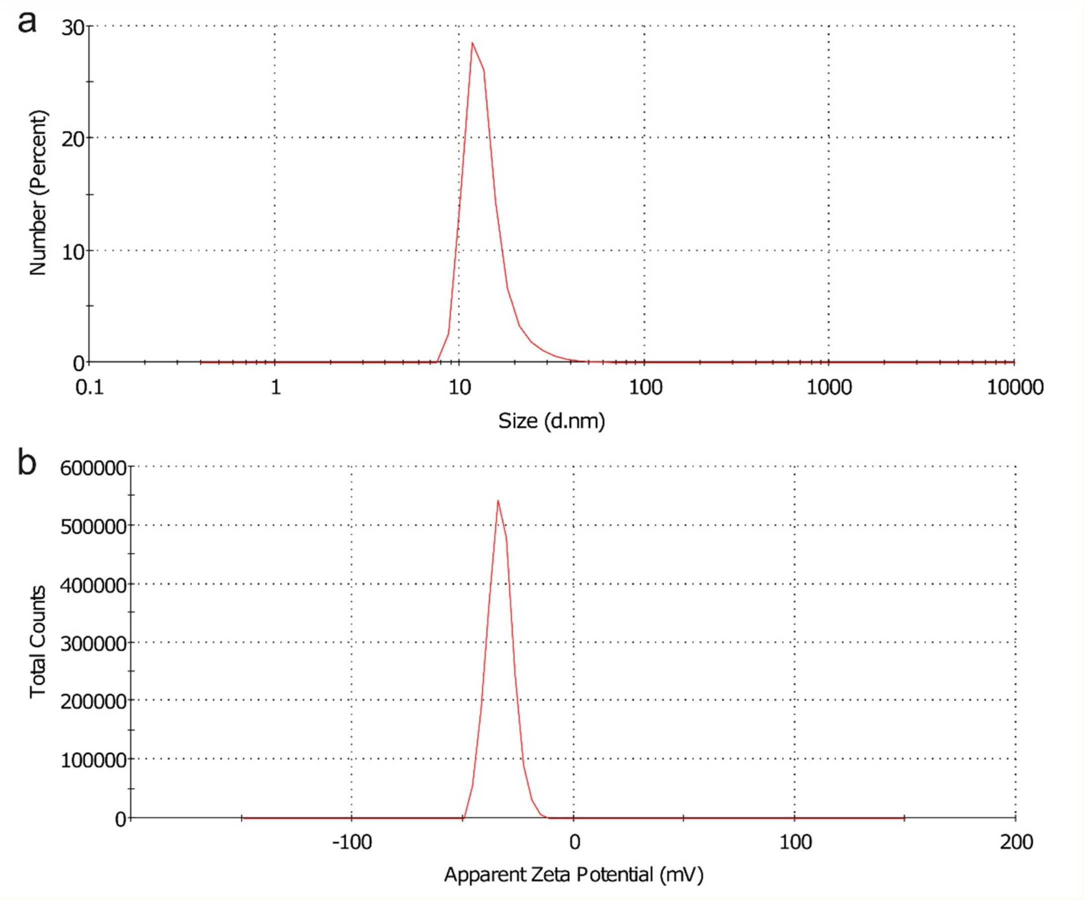

Figure. S1. a) Magnetite nanoparticle size distribution measured by dynamic light-scattering (DLS); b) Zeta potential distribution of magnetite nanoparticles measured by DLS.

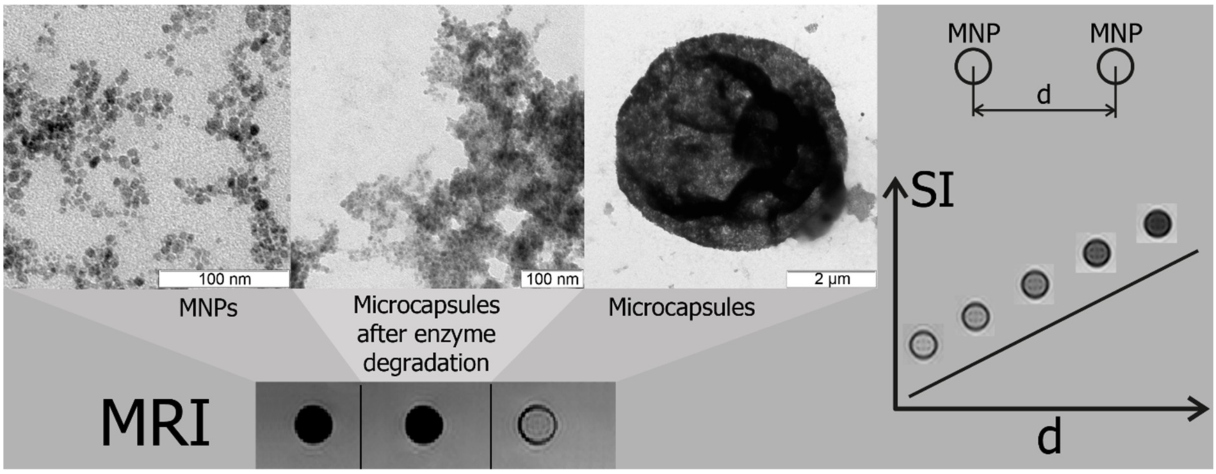

Figure. S2. Dependence of magnetic resonance imaging (MRI) contrast on interparticle distance d, illustrated by transmission electron microscopy (TEM) images of magnetite nanoparticles (MNPs), microcapsules after enzyme degradation and initial microcapsules.

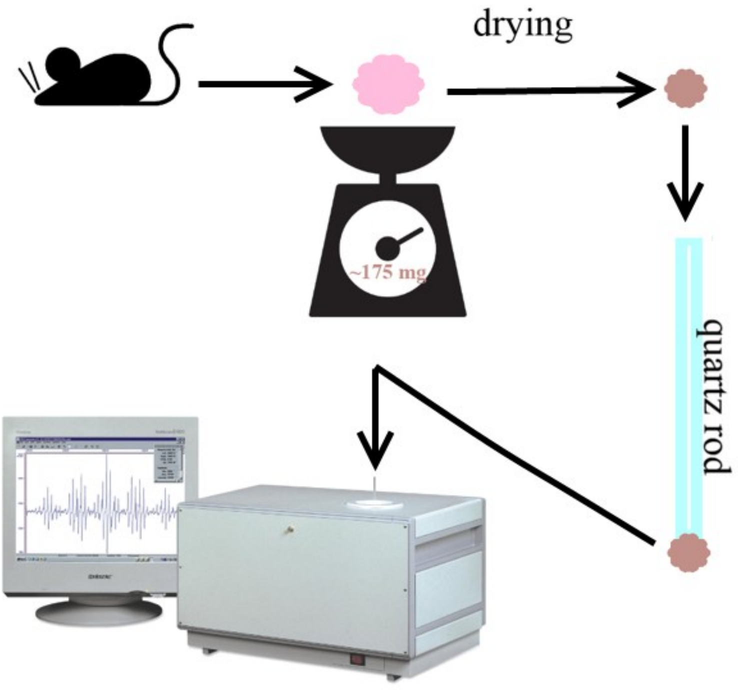

Figure. S3. The sample preparation process for electron spin resonance (ESR) spectroscopy

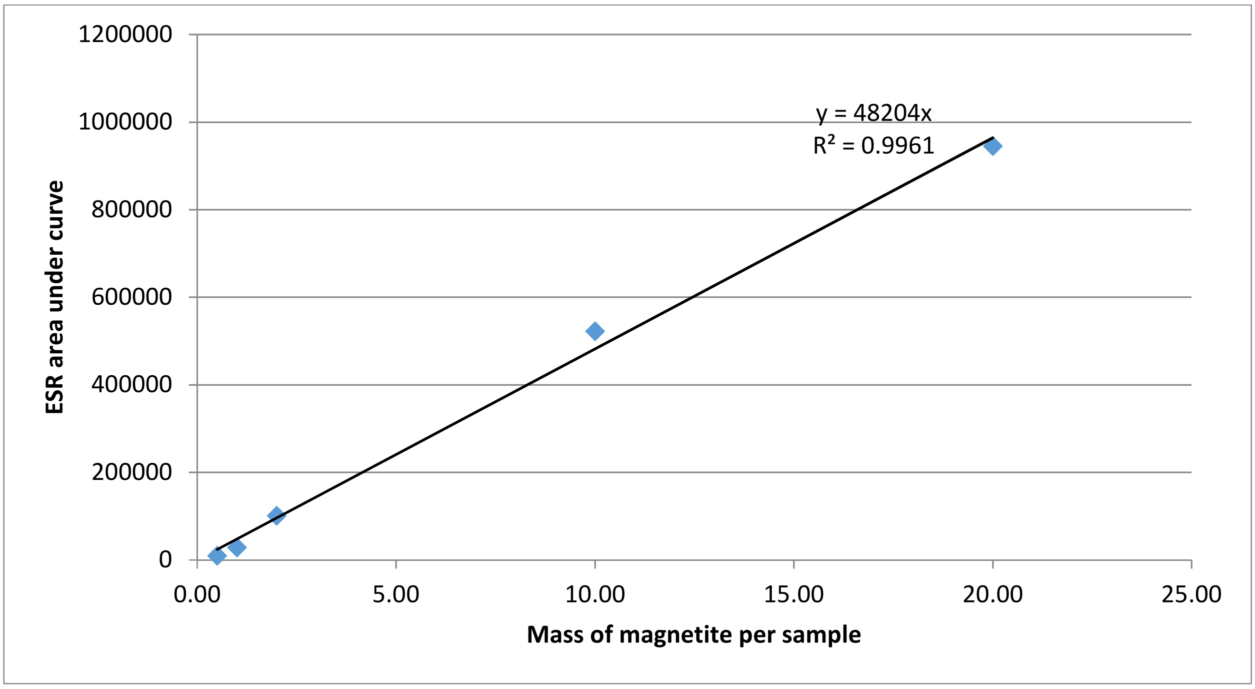

Figure. S4. Chart for mass of magnetite – ESR area under curve dependence

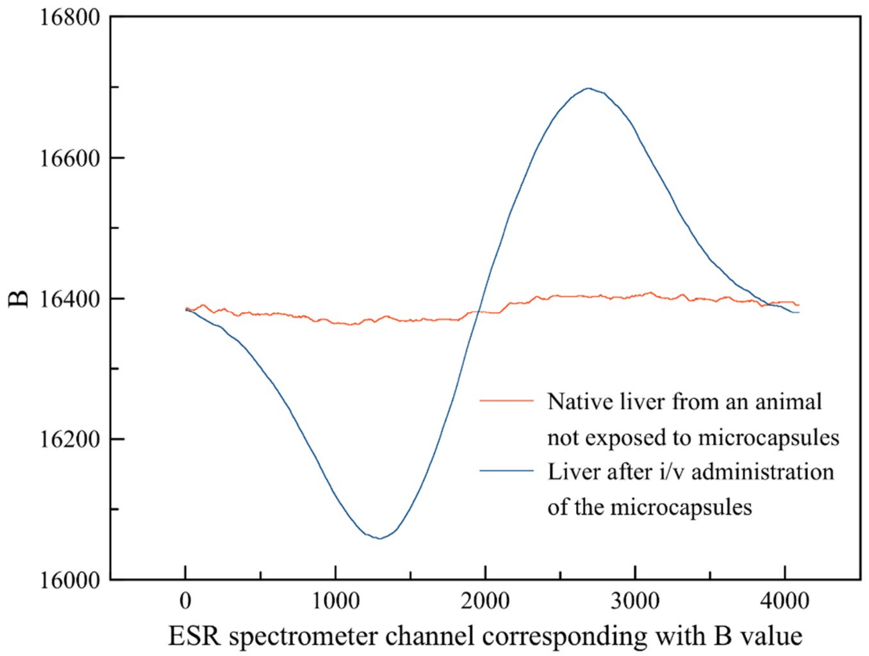

Figure. S5. Comparison of ESR spectra of liver from animals exposed and not exposed to microcapsules

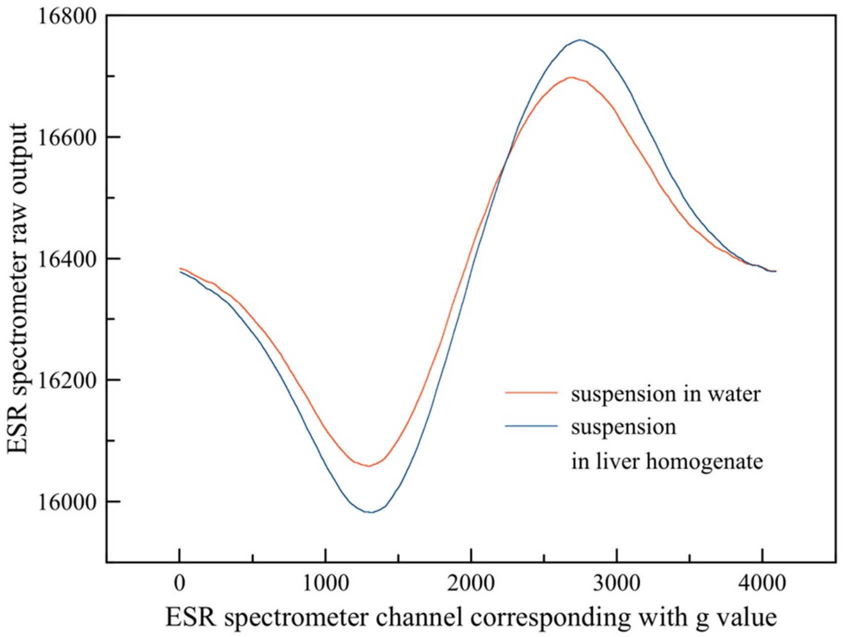

Figure. S6. Comparison of ESR spectra of microcapsules suspension in the water and liver homogenate
